# Heparin-induced thrombocytopenia following Shiga-toxin-associated hemolytic uremic syndrome: a case report

**DOI:** 10.1186/s13256-022-03627-w

**Published:** 2022-10-23

**Authors:** Elia Rigamonti, Tecla Bonora, Mariangela Ventresca, Pietro Cippà

**Affiliations:** 1 Department of Medicine, Division of Internal Medicine, Ospedale Regionale di Lugano, Ente Ospedaliero Cantonale, Bellinzona, Switzerland; 2 Department of Medicine, Division of Nephrology, Ospedale Regionale di Lugano, Ente Ospedaliero Cantonale, Bellinzona, Switzerland

**Keywords:** Hemolytic uremic syndrome, Heparin-induced thrombocytopenia, Differential diagnoses, Shiga toxin, Case report

## Abstract

**Background:**

Up to 50% of cases of Shiga-toxin-producing *Escherichia coli* hemolytic uremic syndrome occur in adults, and the clinical presentation is variable. Microbiological analyses must be performed in all patients with thrombotic microangiopathy to identify Shiga-toxin-producing *Escherichia coli*, even in the absence of diarrhea.

**Case presentation:**

A 79-year-old Caucasian woman was admitted to hospital because of severe proctitis. In the following days, the patient’s level of consciousness declined, and she developed acute kidney injury, thrombocytopenia, and hemolytic anemia. Shiga-toxin-producing *Escherichia coli* was found in fecal cultures, suggesting the diagnosis of hemolytic uremic syndrome. In the following days, her clinical conditions improved, but thrombocytopenia worsened, and the patient developed posterior tibial vein thrombosis. The discordant evolution of thrombocytopenia compared with other clinical and laboratory parameters prompted a new evaluation of its causes. Diagnosis of heparin-induced thrombocytopenia was confirmed by heparin-induced platelet aggregation assay and positive antibodies to platelet factor 4.

**Conclusions:**

A discordant evolution of platelet count in patients with thrombotic microangiopathy requires a systematic reevaluation of the thrombocytopenia.

## Background

Hemolytic uremic syndrome (HUS) associated with Shiga-toxin-producing *Escherichia coli* (STEC HUS) is the most common form of HUS, with 50% of cases occurring in adults [1–3]. The classical clinical manifestation is diarrhea (bloody in about 60% of cases), followed by thrombotic microangiopathy (TMA) causing hemolytic anemia, thrombocytopenia, and acute kidney injury (AKI). STEC HUS can occur without diarrhea, so a rectal swab might be necessary to diagnose STEC infection in some cases. Other causes of TMA must be considered in the diagnostic evaluation, and screening for primary atypical HUS is recommended if STEC HUS and thrombotic thrombocytopenic purpura are excluded. Treatment of STEC HUS is mainly supportive. Close clinical monitoring is required to prevent and treat potential complications. Volume replacement, renal replacement therapy, and circulatory support might be required. Blood transfusions are indicated for symptomatic anemia, whereas platelet transfusions should be avoided. Prognosis of STEC HUS is generally favorable: 70% of patients fully recover, 30% will have renal sequelae, and 4% have long-term neurological complications [4]. Risk factors for a poor prognosis include age < 2 and > 60 years, oliguria or need for dialysis at presentation, and high white blood cell count. The use of antibiotics and antidiarrheals has long been suspected to be associated with adverse outcome; they can lead to drug-induced TTP and toxin release from destruction of bacterial membranes, increasing HUS risk [5].

## Case presentation

**Fig. 1:**
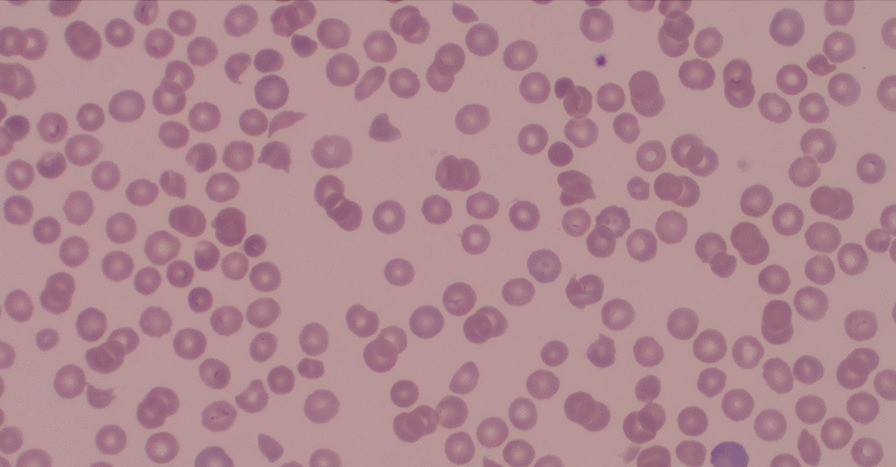
Colonoscopy showing severe proctitis with ulceration of the mucosa

A 79-year-old Caucasian woman with arterial hypertension and type 2 diabetes mellitus was admitted to the hospital because of severe anal pain and constipation. She also suffered from depression after the recent death of her husband. Abdominal examination was unremarkable, while she presented with rectal tenesmus and pain on defecation. At the very beginning, she had hematochezia and mucus leakage, while in the following days she developed severe constipation. Endoscopy and endorectal echography confirmed the clinical suspicion of a severe proctitis (Fig. 1). Upon admission, platelet count was already very low (30 × 10^9^), and for this reason no antithrombotic prophylaxis was given. Local anti-inflammatory treatment was prescribed. Three days later, her clinical condition deteriorated, with the patient appearing confused without focal neurological deficits.

**Fig. 2:**
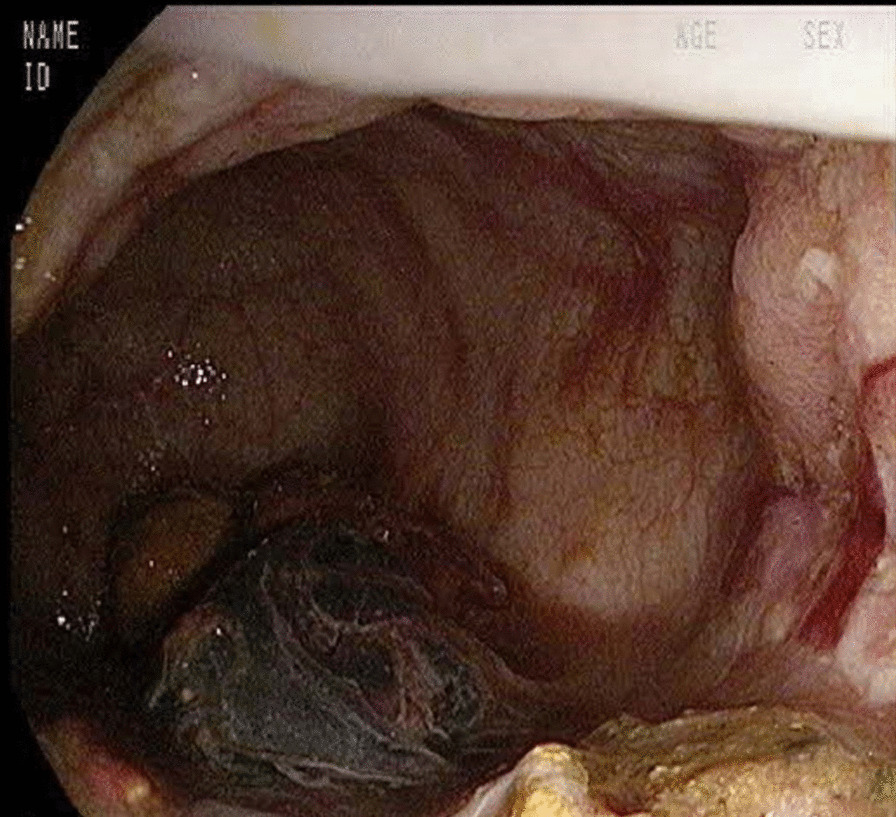
Blood smear at diagnosis: polychromasia. Schistocytes and microspherocytes, suggestive of microangiopathic hemolytic anemia

Blood tests revealed acute kidney injury [AKI stage 2 according to Kidney Disease Improving Global Outcomes (KDIGO), creatinine 251 μmol/L], severe thrombocytopenia (10 × 10^9^/L), and hemolytic anemia [hemoglobin (Hb) 71 g/L, lactate dehydrogenase (LDH) 8618 U/L, negative Coombs test, schistocytes in blood smear; Fig. 2]. The clinical presentation was consistent with TMA. Since thrombotic thrombocytopenic purpura (TTP) could initially not be excluded, we administered high-dose corticosteroids and started plasmapheresis [6, 7]. As soon as ADAMTS13 levels turned out to be normal, plasmapheresis was stopped, and corticosteroid treatment was rapidly reduced and stopped. Serological screening for autoimmune diseases was negative (antinuclear antibody (ANA), antineutrophil cytoplasmic antibody (ANCA), extractable nuclear antigen (ENA) screen, and antibodies associated with anti-phospholipid syndrome). Drug-induced HUS was excluded since no drugs were identified known to cause drug-induced TTP. Complement and anti-factor H activity were within normal limits. Imaging, which encompassed cerebral, abdominal computed tomography (CT) scan, and chest X-ray did not reveal any lesion evocative for neoplasm or alternative explanations for the neurological symptoms. The patient was up to date on breast and uterine cancer screenings, and a full colonoscopy was performed. Microbiological analysis, including viral serologies and repeated blood cultures, was negative. Fecal cultures were positive for Shiga-toxin-producing *Escherichia coli*. Thus, despite the initial atypical presentation with proctitis, the patient had STEC HUS.

**Fig. 3:**
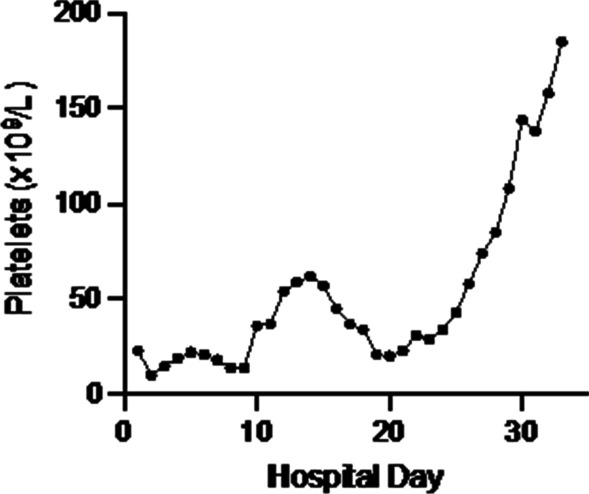
Platelet counts over time

Supportive care was implemented with careful volume management to control AKI, and red blood cell transfusions were required to maintain a hemoglobin level > 80 g/L. In the following days, her clinical condition progressively improved with complete regression of the neurological symptoms, renal function fully recovered, and hemoglobin and platelet counts improved (hemoglobin 93 g/L, thrombocytes 62 × 10^9^/L, creatinine 206 μmol/L). A few days later (day 15 of hospitalization, Fig. 3), we observed a new deterioration of the platelet count (thrombocytes 20 × 10^9^/L) and the patient developed posterior tibial vein thrombosis despite prophylactic doses of heparin. The case was reevaluated because of the unexpected clinical evolution: several differential diagnoses were considered, including a relapse of STEC HUS or drug-induced immune thrombocytopenia.

Moreover, post-transfusion purpura was considered as an alternative diagnosis [8], but was not a major concern because red blood cell units transfused were washed to avoid exposure to platelet membranes. Since heparin-induced thrombocytopenia (HIT) was among the top differential diagnosis (4Ts preclinical score of 6 points), heparin was switched to argatroban. Antibodies to platelet factor 4 turned out to be positive, and heparin-induced platelet aggregation (HIPA) assay confirmed the diagnosis of HIT. Argatroban was continued for a total of 4 weeks, and afterwards we switched to apixaban according to current guidelines [9].

Anticoagulation management in this patient with STEC HUS followed by HIT was challenging, since the patient presented several episodes of rectal hemorrhage in the context of persistent proctitis, requiring transfusion of 12 erythrocyte concentrates. The final outcome was favorable: the patient was discharged in good clinical condition, with normal platelet count and neurological function. At the ambulatory follow-up visit 3 months later, the patient was independent at home, renal function had recovered, and the hematological parameters were normal. The lower-limbs ultrasound documented full resolution of deep vein thrombosis, and anticoagulation was stopped.

## Discussion and conclusions

This case presented a combination of challenges. First, the presentation of STEC HUS was not typical: the majority of cases occur in children or young adults and only 14% in those aged ≥ 60 years [10]. The classical clinical manifestation is diarrhea, whereas our patient presented with severe proctitis. This reinforces the recommendation to perform fecal analysis or rectal swab in all patients with TMA, independently of the initial clinical manifestation [11].

Second, the patient presented with a thrombocytopenia relapse 2 weeks after hospitalization. Cases of relapsing HUS after STEC HUS related to defective complement or concomitant Parvovirus B19 infection were recently reported by Schwarz *et al*. [12]. In contrast, in our case only thrombocytopenia was deteriorating again after initial improvement. This required to broaden the differential diagnosis and to reconsider other causes of thrombocytopenia, including HIT.

Third, the management of anticoagulation in a patient with STEC HUS followed by HIT deep vein thrombosis and recurrent rectal hemorrhage in the context of a severe proctitis required special attention. The association of STEC HUS and HIT might be a pure coincidence, considering that HIT has been reported in up to 5% of patients exposed to heparin for more than 4 days [13], but it is tempting to consider a potential causal link between the causes of thrombocytopenia. Experimental studies indicated that anti-PF4 antibodies might occur in response to negatively charged polysaccharides present on the surface of the bacteria mimicking the PF4–heparin complex [14]. Furthermore, HUS can lead to platelet activation and PF4 release in a highly inflammatory environment, which might trigger the production of anti-PF4 antibodies, as previously suggested [15].

## Data Availability

The data used in the case report are available on reasonable request.
